# Clinical trial considerations on male contraception and collection of pregnancy information from female partners

**DOI:** 10.1186/1479-5876-10-129

**Published:** 2012-06-21

**Authors:** Maria Longauer Banholzer, Heinrich Buergin, Christoph Wandel, Georg Schmitt, Elmar Gocke, Richard Peck, Thomas Singer, Theresa Reynolds, Marie Mannino, Jonathan Deutsch, Lucette Doessegger

**Affiliations:** 1 Safety Risk Management, Licensing & Early Development, F. Hoffmann-La Roche AG Ltd, Basel, CH, Switzerland; 2 Safety Risk Management, Licensing & Early Development, F. Hoffmann-La Roche Ltd, Nutley, NJ, USA; 3 Pharma Research & Early Development, Non-Clinical Safety, F. Hoffmann-La Roche, Toxicology Department, Basel, CH, Switzerland; 4 Clinical Pharmacology Department, Roche Products, Welwyn, UK; 5 Pre-Clinical Safety Department, Genentech, Inc, South San Francisco, CA, USA; 6 Medical Safety Assesssment, Janssen Research & Development, LLC, Raritan, NJ, USA

**Keywords:** Genotoxicity, Teratogenicity, Male contraception, Paternal exposure

## Abstract

**Background:**

There is little guidance regarding the risk of exposure of pregnant women/ women of childbearing potential to genotoxic or teratogenic compounds via vaginal dose delivered through seminal fluid during sexual intercourse.

**Method:**

We summarize current thinking and provide clinical trial considerations for a consistent approach to contraception for males exposed to genotoxic and/or teratogenic compounds or to compounds of unknown teratogenicity, and for collection of pregnancy data from their female partners.

**Results:**

Where toxicity testing demonstrates genotoxic potential, condom use is required during exposure and for 5 terminal plasma half-lives plus 74 days (one human spermatogenesis cycle) to avoid conception.

For non-genotoxic small molecules and immunoglobulins with unknown teratogenic potential or without a no observed adverse effect level (NOAEL) from embryo-fetal development (EFD) studies and no minimal anticipated biological effect level (MABEL), condom use is recommended for males with pregnant partner/female partner of childbearing potential. For teratogenic small molecules with estimated seminal fluid concentration and a margin between projected maternal area under the curve (AUC) and NOAEL AUC from EFD studies of ≥300 (≥100 for immunoglobulins) or in the absence of a NOAEL with a margin between MABEL plasma concentration and maternal C_max_ of ≥300 (≥10 for immunoglobulins), condom use is not required. However, condom use is required for margins below the thresholds previously indicated. For small molecules with available seminal fluid concentrations, condom use is required if margins are <100 instead of <300. Condom use should continue for as long as the projected margin is at or above the defined thresholds.

Pregnancy data should be proactively collected if pregnancy occurs during the condom use period required for males exposed to first-in-class molecules or to molecules with a target/class shown to be teratogenic, embryotoxic or fetotoxic in human or preclinical experiments.

**Conclusion:**

These recommendations, based on a precaution principle, provide a consistent approach for minimizing the risk of embryo-fetal exposure to potentially harmful drugs during pregnancy of female partners of males in clinical trials. Proactive targeted collection of pregnancy information from female partners should help determine the teratogenic potential of a drug and minimize background noise and ethical/logistical issues.

## Background

Genotoxic molecules may cause DNA damage or impairment of chromosome replication, which may be passed on to progeny at conception when damage occurs to the genetic material of germ cells. Depending on the type (e.g., gene mutations, chromosomal aberrations, chromosomal misdistributions) and location of the damage, genetic alterations may lead to embryo-fetal death, birth defects or hereditary defects.

Teratogenic molecules may affect embryogenesis and fetal development, leading to birth defects. Birth defects are thought to account for more than 20% of infant deaths [1], and approximately 10% of birth defects are caused by teratogens [2].

Regulatory guidance and instructions in local package inserts address restriction of the use of genotoxic and teratogenic compounds in women of childbearing potential (WOCBP) and pregnant women. However, there is a lack of consistency in labeling documents regarding the use of contraception in men who take genotoxic pharmaceuticals. Fathering a child should be prevented during and for an appropriate period of time after dosing with genotoxic molecules.

Guidelines exist on the collection of pregnancy data from the female partners of males exposed to molecules that may be mutagenic or teratogenic [3]. However, there are few guidances on the risk of exposure of pregnant women or WOCBP to teratogenic pharmaceuticals via a dose delivered through seminal fluid during sexual intercourse that is absorbed into the maternal circulation. The risk of exposure of the embryo or fetus resulting from an unintended vaginal dose of a medication can be limited by taking precautions with condom use. There are no clear guidelines specifying what levels of a teratogenic pharmaceutical constitute a risk to embryo-fetal development and when precautions should be taken for men exposed to molecules of either known or unknown teratogenicity.

## Methods

We summarize the current scientific understanding and provide recommendations for condom use for males participating in clinical trials who have reached puberty and are sexually active with a female partner of childbearing age, and who are exposed to genotoxic or teratogenic compounds or to compounds of unknown teratogenicity. We also summarize guidelines for proactive collection of pregnancy data in clinical trials from female partners of males exposed to potentially genotoxic or teratogenic molecules.

### Drugs in seminal fluid

The 3 mechanisms for exposure of a conceptus to chemicals in semen have been summarized by Klemmt and Scialli [4] and are presented in this section. 

1. Access of chemicals to the maternal circulation after absorption from the vagina, at any time pre or post conception. Vaginal absorption of chemicals in humans can be demonstrated however the degree of this absorption depends on the physico-chemical properties of the molecule. While no studies are available on the systemic absorption of pharmaceutical products delivered in semen to the human vagina, there are studies on the systemic absorption of pharmaceutical products delivered vaginally to non-pregnant women. It does not appear that vaginal exposure to chemicals in the semen would be quantitatively important. To use a rough example, clindamycin has the highest semen:blood ratio of medicinals tested (11:3). If a total volume of semen containing clindamycin was absorbed vaginally, the final expected blood exposure of a woman has been calculated as three orders of magnitude lower than the blood exposure of the man who produced the semen. Direct chemical exposure of the conceptus following transport from the vagina to the uterine cavity.

2. Direct delivery of chemicals from the semen through the cervical mucus into the uterine cavity of humans has not been demonstrated, although it may occur in species (e.g. rats) where the seminal fluid has access to the uterine cavity.

3. Delivery of chemical bound to the sperm cell to the egg and subsequent conceptus. There is also the possibility that chemical binding to spermatozoa could result in embryo-fetal exposure, since chemicals in or on human cells have been demonstrated with tetracycline and cocaine *in vitro* and aluminium, lead and cadmium *in vivo*. An *in vitro* study by Yazigi *et al* investigated the interaction of cocaine with human spermatozoa [5]. The motility and viability, but not fertilizing ability, of sperm bound to cocaine was investigated. Using this study, oocyte concentrations would be approximately 5 orders of magnitude lower than blood concentrations associated with cocaine abuse.

Even though the transmission of a medicinal product administered to a male to the conceptus appears to be low, in the absence of clinical evidence and with limited preclinical data, risk mitigation procedures for clinical trials should be considered as outlined in the following sections.

### Preclinical testing

#### Genotoxicity testing

Before entry into humans, generally a comprehensive assessment of the genotoxic potential of small molecule drug candidates is performed via a number of complementary tests. The standard battery for small molecule genotoxicity testing includes (1) a test for gene mutation in bacteria (Ames test), (2) an *in vitro* cytogenic test for chromosomal damage (chromosome aberration [CA], micronucleus test [MNT]) or mouse lymphoma thymidine kinase (tk) gene mutation assay and (3) an *in vivo* test for chromosomal damage in rodent hematopoietic cells (e.g., the MNT *in vivo*).

The Ames test examines a compound by observing whether it can revert mutated strains of Salmonella typhimurium bacteria which are auxotrophic for histidine biosynthesis, thus allowing them to grow on histidine-free medium [6-8]. A standard set of different Salmonella strains that detect different types of mutations (e.g., base substitutions or frameshift point mutations) is used.

Gross chromosomal damages in mammalian cells are tested by the CA assay in human lymphocytes or established cell lines, where structural damage is detected by microscopic scoring of chromosomes in mitotic metaphase cells [9]. The mouse lymphoma tk gene mutation assay can test for mutations at specific loci, such as the tk gene, and allows for the detection of mutations that are not detected in bacterial systems [10]. Furthermore, the MNT can be applied in genotoxicity testing in mammalian cells *in vitro*. The assay is based upon the examination of micronuclei that are formed due to chromosomal fragments and are located outside of the normal cell nucleus [11].

The MNT *in vivo* is the preferred *in vivo* test for the determination of the chromosomal damage in rodent hematopoietic cells and is performed in bone marrow or peripheral blood. The determination of micronuclei in peripheral blood has now been adapted for measurement by flow cytometry [12]. Additionally, primary DNA damage induced by a compound is detected by single cell gel electrophoresis (the comet assay), which involves electrophoresis of nucleoids embedded into agarose followed by DNA staining to examine DNA integrity [13].

The impact of positive pre-clinical findings during the development of a compound depends on the indication and the duration of treatment. Genotoxic compounds are not tested in healthy volunteers. The battery of assays mentioned previously has a high sensitivity; hence, development of a small molecule compound that was found to be genotoxic would be stopped unless the benefits outweighed the risk or if there was convincing evidence that the findings were irrelevant for human exposure. There are no regulatory requirements for genotoxicity testing of biopharmaceuticals because, based on their physicochemical properties, these large molecules are not expected to possess DNA damaging properties. Although testing may be considered for molecules that interfere with DNA synthesis or contain non-natural chemical constituents, biopharmaceuticals are typically not tested for genotoxicity because the standard testing battery is not relevant.

#### Embryo-fetal development testing

Teratogenicity of small molecules may also be tested using *in vivo* and *in vitro* methods. As an *in vitro* screen, the embryonic stem cell test (EST) may be performed before entry into humans or before screening for clinical candidates; however, the EST currently does not have regulatory acceptance and alone is not sufficient to allow clinical trials in WOCBP. *In vivo* testing may be required, depending on the indication and potential impact on the label, and is generally performed in two species (one of which should be a non-rodent); a positive result from one species is sufficient to define teratogenicity [14].

There are currently no established *in vitro* assays to test for the teratogenicity of larger biopharmaceuticals, such as monoclonal antibodies and fusion proteins. The *in vivo* testing of biopharmaceuticals is performed on a case-by-case basis. For biopharmaceuticals, a scientific rationale for the testing strategy is developed based on drug target or pathway, data from systemic toxicity studies and the biological functional relevance of available animal models.

#### Special considerations in testing

When the mechanism of action of a drug results in a genotoxic and/or teratogenic effect (e.g. microtubule polymerase inhibitor), reproductive toxicity studies may not be conducted.

### Regulatory considerations for preclinical testing to support drug development

#### Genotoxicity testing and EFD studies

For entry into humans, for small molecules, generally the Ames test is required for single dose clinical trials; in addition, the CA assay is needed to support multiple dose clinical development trials. Preliminary embryo-fetal development studies (teratogenicity studies) in two species may be available, depending on indication and potential impact on label [14]. For monoclonal antibodies generally no studies are performed for assessing genotoxicity and teratogenicity.

For entry of a small molecule drug into Phase II clinical trials, preliminary EFD studies may be performed to assess the embryotoxic potential. Depending on mode of action and clinical study design (e.g., patient population, duration of treatment, planned number of WOCBP to be included and indication), full embryo-fetal development studies in two species, conducted according to good laboratory practice (GLP) or high-quality scientific standards, may occasionally be needed [14].

For Phase III trials involving small molecules, standard regulatory *in vivo* EFD studies may be required in two species, according to GLP, depending on indication. For monoclonal antibodies, an EFD study in one responder species or an extended pre- and post-natal study (ePPND) in a non-human primate may be sufficient and may be performed during Phase III trials [14-16].

#### Margin considerations in current regulatory environment

For a teratogenic small molecule, a margin of 1000 between the extrapolated plasma area under the curve (AUC) in the female partner caused by a potential vaginal dose and the measured plasma AUC at the no observed adverse effect level (NOAEL) in EFD studies has been used previously (personal communication). Considerations on therapeutic index between toxic reproductive dose response in 10% of a sensitive species and efficacious dose in 90% of the test species were included in a draft US guidance issued in 2001 [17].

Recently, for an antibody with no teratogenicity data available, a margin of approximately 3,000 between estimated fetal exposure (10% of maternal exposure) and projected therapeutic exposure was accepted by some regulators to obtain a waiver for condom use.

### Regulations for male contraceptive measures and pregnancy data collection from pregnant female partner

#### Prior to new drug application

At the present time, there are no standard guidelines on male contraceptive requirements or proactive collection of pregnancy data from female partners of males exposed to potentially genotoxic and/or teratogenic compounds during the conduct of clinical trials. The risk of unintentional exposure of the embryo/fetus to potentially harmful medicinal products can be minimized by using highly effective birth control methods (i.e., resulting in a low failure rate [less than 1% per year]) [3,18]. When indicated (e.g., trials involving drugs which are potentially mutagenic, or toxic to the reproductive system), an appropriate contraception provision should be included in the trial in accordance with the International Conference on Harmonisation (ICH) guidelines [19]. Clinical guidance states that drug protocols should stipulate the use of condoms for the duration of the study and for a suitable time period after the last drug dose (e.g., 5 half-lives) to ensure the embryo/fetus is not exposed to the drug through vaginal absorption [19,20].

Individual cases of serious unexpected adverse reactions associated with a medicinal product should be reported on an expedited basis (reported immediately and within 15 days of receipt) [3,18,21]. These reporting timelines also apply to embryo-fetal anomalies, fetal deaths, spontaneous abortions and serious adverse reactions in the neonate or infant.

#### Peri/post-new drug application

The focus of this document is to provide recommendations for clinical trials; however, guidelines for marketed products are also outlined to provide a more comprehensive overview.

The marketing authorization holder (MAH) is responsible for reporting congenital anomalies on an expedited basis if the embryo-fetus was exposed to a medicinal product [18,21]. It is also important to report ‘normal’ pregnancy outcomes following drug exposure in the periodic safety update reports (PSURs) to help deduce the teratogenic potential of a drug [3,21].

Guidelines state that for teratogenic medicinal products, the MAH must develop appropriate measures of active surveillance defined within a risk management plan (RMP) [3,18,21].

Pregnancy exposure registries are prospective, observational studies that actively collect information on drug exposure during pregnancy. The US Food and Drug Administration (FDA) regulations recommend registries in cases of medicinal products with the potential to cause harm during pregnancy and when inadvertent drug exposure to WOCBP is likely to be common [22,23]. Registries can be initiated at any time and sponsors may be asked to commit to conducting a post-authorization exposure registry before approval is given [23]. They may also include pregnancy outcomes from female partners of males exposed to drugs (paternal exposure).

#### Male contraceptive methods

The ICH guidelines specify that methods of birth control that are highly effective have a failure rate of less than 1% when used consistently and correctly, and the Medicines and Healthcare Products Regulatory Agency (MHRA) specifies that acceptable forms of male contraception for clinical trials include sexual abstinence, vasectomy and barrier methods [20]. In addition to sexual abstinence, the FDA recognizes sterilization as one of the most effective methods of birth control [24].

In certain circumstances, e.g., for highly teratogenic compounds, two forms of contraception are required.

### Translation of preclinical findings

#### Assumptions made to estimate vaginal dose

Current guidelines for teratogenic compounds apply to drugs that have been shown to be teratogenic in at least one animal species or have not been tested. The potential absorption of teratogenic compounds into the maternal circulation takes into account the concentration of the molecule in seminal fluid. When the concentration of drug in seminal fluid is unknown a number of assumptions can be made: the volume of seminal fluid is assumed to be 6 mL. For small molecules the drug concentration in seminal fluid is assumed to be equal to the concentration of the molecule in paternal plasma and 100% thereof is assumed to be absorbed by the vaginal mucosa and cervix (referred to as the ‘vaginal dose’). For monoclonal antibodies, the seminal fluid concentration is assumed to be 1% of the paternal plasma concentration [25] and the vaginal dose is 10% of this [26,27]. For both types of molecule, pharmacokinetic linearity is assumed for extrapolation to the projected maternal AUC associated with vaginal dose. If human pharmacokinetic (PK) data are not available, a drug metabolism and pharmacokinetics (DMPK) model should be used and PK variability should be taken into account (Table 1). For some molecules, seminal fluid concentrations may exceed time-matched plasma concentrations (e.g., lenalidomide); [28] however, adequate protection is thought to be provided by the safety margins described in this review.

**Table 1:** Assumptions for estimation of female partner AUC associated with vaginal dose

	**Small molecules**	**Monoclonal antibodies**
Seminal fluid concentration	C_max_	1% of C_max_
Seminal fluid volume	6 mL	6 mL
Vaginal absorption	100%	10%
Placental transfer	100%	10% (first trimester)
PK linearity	Yes	Yes
First pass effect	No	N/A
High protein binding	Correction for difference in fraction unbound between humans and animals tested	N/A
PK variability	Yes	Yes
Extrapolate from human PK if available; otherwise use DMPK model		

C_max_, maximum concentration; PK, pharmacokinetic; DMPK, drug metabolism and pharmacokinetics.

For small molecules that are highly protein-bound, a correction for the differences in fraction unbound between humans and animals tested should be made. The ratio of maternal to embryo-fetal concentrations of the molecule is assumed to be similar across species, including humans. For small molecules, it is also assumed that placental transfer is 100%. For monoclonal antibodies it is assumed that embryonic immunoglobulin G (IgG) concentrations are up to 10% of maternal IgG concentrations during the first trimester (Table 1); [26,27] which is the most critical period in human development, and corresponds to the sensitive period for structural birth defects during intrauterine development. However, at term, IgG in the fetal circulation may be as high as 1.8 times the maternal level.

#### Minimal anticipated biological effect level (MABEL) approach

The MABEL is the anticipated dose level leading to a minimal biological effect level in humans. The MABEL calculation should use all relevant and available *in vitro* and *in vivo* information from PK and pharmacodynamic (PD) data such as: [29]

· Receptor binding and occupancy studies, e.g., *in vitro* in target cells from human and relevant animal species and *in vivo* in the relevant animal species.

· Concentration-response curves *in vitro* in target cells from human and the relevant animal species and dose response *in vivo* in the relevant animal species.

· Exposures at pharmacological doses in the relevant species.

These data should be integrated into a PK/PD modeling approach to enable calculation of the MABEL. There are several prerequisites for using MABEL, including: no off-target effect (including metabolites) and no toxicities associated with pharmaceutical properties (e.g., drug deposition in bradytrophic tissues such as bone). When these prerequisites are taken into account, it is unlikely that a concentration of drug that does not cause biological effects would produce teratogenicity.

The MABEL can be considered for the margin calculation against maternal C_max_ via exposure through seminal fluid for molecules with no NOAEL from embryo-fetal development studies, or for those in which no embryo-fetal development studies are available.

## Results and discussion

### Impact of preclinical findings on protocol eligibility criteria for males and on collection of pregnancy information from their female partners

#### Genotoxic compounds: Condom use for avoiding conception

A potential risk for birth defects due to DNA damage in the germ cells of the father is conceivable. However, toxicological and clinical experience shows that an increase of birth abnormalities above the background rate is only seen with the most potent genotoxins at very high dose levels, e.g., cyclophosphamide (Cytoxan®).

**Figure 1:**
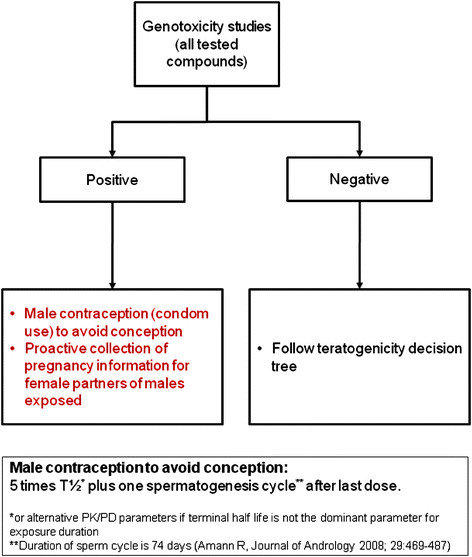
**Genotoxicity Decision Tree.** *Highly effective forms of contraception result in a failure rate of less than 1% per year and include implants, injectables, combined oral contraceptives, some intrauterine devices, sexual abstinence or vasectomy.[31]

If toxicity testing demonstrates that a molecule has genotoxic potential, condom use is required to avoid conception. Condom use should continue for 5 terminal plasma half-lives for small molecules plus 74 days, the duration of the human spermatogenesis cycle [30]. In the event of pregnancy in the female partner, pregnancy data should be collected after obtaining informed consent from the female partner (Figure 1).

#### Teratogenic compounds: Condom use for avoiding vaginal dose

The potential risk to progeny from teratogenic compound exposure via the vaginal dose is considered to be low since the transfer of a drug via seminal fluid to the embryo-fetus is minor. Generally there is no indication in US package inserts for non-genotoxic teratogens about the use of condoms in males with a pregnant female partner or a WOCBP. An exception with a specific indication of no measures includes isotretinoin [Accutane®, Roche] for which no measures are substantiated by very low levels in seminal fluid, and where the vaginal dose is estimated at a million times lower than the 40 mg oral dose taken by males [32]. The use of condoms is, however, required for thalidomide and genotoxic teratogens such as ganciclovir. Further labeling regarding condom use recommendations may be country specific.

**Figure 2:**
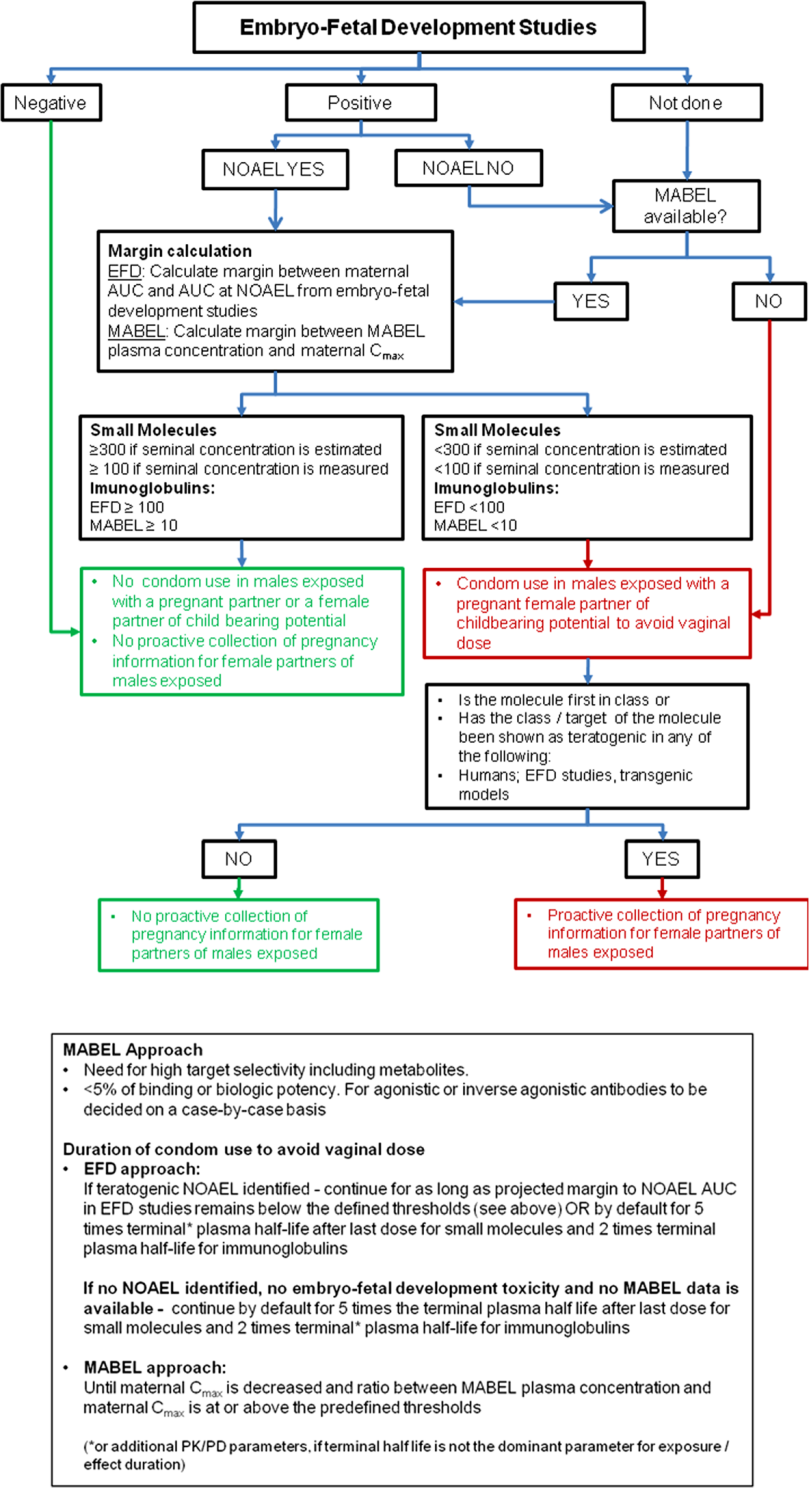
Teratogenicity Decision Tree.

In clinical development, condom use is a precautionary measure for patient progeny and is broadly applied for molecules with unknown teratogenicity. To decide whether condom use is necessary for male patients taking a new molecule, the potential teratogenic risk of the compound should be evaluated (Figure 2). The teratogenicity decision tree is applicable to non-genotoxic compounds or if the genotoxicity test battery is not required.

If experiments on embryo-fetal development are available and the test molecule is not teratogenic then condom use and collection of pregnancy information for female partners of exposed males is not necessary (unless the test molecule is genotoxic).

If a non-genotoxic small molecule or a monoclonal antibody is known to be teratogenic from EFD studies and, the margin between maternal AUC in humans and AUC at NOAEL in the most sensitive animal species is ≥300 when seminal concentration is estimated (≥100 when seminal concentration is measured and for immunoglobulins), condom use is not required. However, if this margin is <300 (<100 when seminal concentration is measured and for immunoglobulins) condom use is considered necessary for as long as the projected margin remains below this threshold, or by default, for 5 terminal^1^ plasma half-lives for small molecules and 2 terminal plasma half-lives for immunoglobulins.

If teratogenicity has been demonstrated in EFD studies and no NOAEL or MABEL are available, condom use is recommended for 5 terminal^[1]^ plasma half-lives for small molecules and 2 terminal^[1]^ plasma half-lives for immunoglobulins. If a MABEL is available and the margin between MABEL plasma concentration and maternal C_max_ is ≥300 when seminal concentration is estimated (≥100 when seminal concentration is measured and ≥10 for immunoglobulins), condom use is not required. However, if the margin is <300 (<100 when seminal concentration is measured and <10 for immunoglobulins), condom use is considered necessary for as long as the projected margin remains below this threshold or for 5 terminal^[1]^ plasma half-lives for small molecules and 2 terminal^[1]^ plasma half-lives for immunoglobulins.

If EFD experiments are not available but the MABEL is known and the margin between MABEL plasma concentration and maternal C_max_ is ≥300 (when seminal concentration is estimated, ≥100 when seminal concentration is measured and ≥10 for immunoglobulins), condom use is not required. However, if the margin is <300 (<100 if seminal concentration is measured and <10 for immunoglobulins), condom use is required for as long as the projected margin remains below these thresholds. In cases where experiments on EFD are not available and the MABEL is not available, condom use is required for 5 terminal^[1]^ plasma half-lives for small molecules and 2 terminal^[1]^ plasma half-lives for immunoglobulins.

When condom use is required, pregnancy information should be collected from female partners becoming pregnant during the period of condom use, if the molecule is first-in-class, or its class or target has been shown or is suspected to be teratogenic (in at least one of the following: humans, EFD studies, transgenic models) [3].

## Conclusions

Guidelines exist on minimizing the risk of exposure of pregnant women to potentially harmful drugs [3,18,22,33]. However, contraceptive requirements for males exposed to medicinal products in clinical trials are handled inconsistently. We present genotoxicity and teratogenicity ‘decision tree’ algorithms which can be applied when assessing contraceptive requirements for males exposed to small molecules or immunoglobulins in clinical trials and the need for data collection of pregnancy information from their female partners. For biopharmaceuticals other than monoclonal antibodies, a case by case approach should be used.

This clinical trial guidance aims at providing risk minimization of embryo-fetal exposure to potentially harmful medicinal products during pregnancy of female partners of males exposed to small molecules and monoclonal antibodies. Targeted collection of pregnancy information from the pregnant partners of males exposed may help to better understand the teratogenic potential of a drug in human.

Once the investigator discusses and communicates the potential risk for the offspring associated with the drug, it is the responsibility of the male exposed to the drug in the context of a clinical trial to inform his female partner(s) of the need to comply with the risk minimization measures. It is understood that the requirement for condom use is applicable to sexually active subjects regardless of sexual practice.

The recommendations are based on a precaution principle using available scientific information and may further evolve.

## Endnotes

^[1]^ Alternative PK/PD parameters should be used if the terminal half-life is not the dominant parameter for exposure duration.

## Competing interests

The authors declare that they have no competing interests.

## Authors’ contributions

MLB: Major contributor to concept and translational aspects of the manuscript. Critical involvement in drafting and revising the manuscript. HB, GS: Major contributors to the preclinical sections and translational aspects**.** EG: Major contributor to the genotoxicity section. TS: Co-Approver of the manuscript. TR: Critical reviewer of the preclinical sections. CW: provided data and major contributor to pharmacological aspects. MM, JD: Provided summary of relevant literature and regulatory guidance. RP: Reviewer of pharmacological aspects. LD: Major contributor to concept and all aspects of the manuscript, provided guidance in writing and revising the manuscript, co-approver. All authors read and approved the final manuscript.
